# Seroprevalence of *Acanthamoeba* Antibodies in Rheumatoid Arthritis Patients by IFAT, Tehran, Iran 2007

**Published:** 2010-03

**Authors:** M Eftekhar, A Athari, A Haghighi, N Mosaffa, F Shahram, A Abadi

**Affiliations:** 1 Dept. of Parasitology, School of Medicine, Shahid Beheshti University, M. C., Tehran, Iran; 2 Dept. of Immunology, Shahid Beheshti University, M. C., Tehran, Iran; 3 Rheumatology Research Center, Tehran University for Medical Sciences, Iran; 4 Dept. of Community & Health, Shahid Beheshti University, M. C., Tehran, Iran

**Keywords:** *Acanthamoeba*, Rheumatoid Arthritis, PCR, IFA, Iran

## Abstract

**Background:**

This preliminary study was conducted to discriminate the prevalence of *Acanthamoeba* antibodies in rheumatoid arthritis (RA) patients and healthy controls to analyze the correlation between these two groups.

**Methods:**

From October 2006 to August 2007 a total of 121 serum samples from RA patients attending the Rheumatolgy Department at Shariati Hospital in Tehran were obtained and stored at -20°C until using by indirect fluorescent-antibody test (IFAT). RA was diagnosed according to the American Collage of Rheumatology classification criteria. The organism used in this study was isolated from various water resources in Tehran, Iran cultured axenically and then went on a PCR assay based on 18S rRNA to identify the genus *Acanthomoeba*. Indirect immunofluorescence antibody (IFA) staining of serum samples was carried out to detect anti *Acanthomoeba* antibodies.

**Results:**

In culture, out of 22 samples, 13(59%) were grown in xenic but only two in axenic medium. PCR amplified a 904bp fragment, specific for *Acanthamoeba*. Of examined serum samples, *Acanthamoeba* antibodies were present in 70 (57.8%) and 52 (41.2%), respectively. The highest titer of antibodies (1:320) was detected in one patient with RA.

**Conclusion:**

Our study supports the hypothesis that some parasitic microorganisms can involve and contribute toward the development of rheumatoid syndromes.

## Introduction

Free living amoebae belong to the genus *Acanthamoeba* are opportunistic protozoa and ubiquitous in the environment including soil, water, air etc (1, 2). Their wide distribution in nature brings humans into contact with these amoebae and there are many evidences showing the presence of antibodies to *Acanthamoeba* in human and animal population (3, 4). The genus *Acanthamoeba* is now well recognized as human pathogen causing serious and life-threatening infections such as granulomatous amebic encephalitis (GAE), a fatal disease of the central nervous system (CNS) and amebic keratitis (AK), a painful sight-threatening disease of the eyes (5). In a study in Iran, *Acanthamoeba* genotype were determined as for 13 keratitis isolates and most of them belonged to T4 and 12 environmental isolates with majority of T2 genotype (6). In another study, out of 80 collected samples from various natural habitats, 46.25% contained *Acanthamoeba* sp. All of the soil samples had shown positive culture in contrast to tap water, and all were negative (7).

Although cases of acanthamoebiasis have been reported from immunocompetent children, but it has been postulated that impairment of host defense mechanisms in immunocompromised, immunosuppressed and debilitated individuals (alcoholics, diabetics, patients with autoimmune diseases) can spread the infection from the primary site to other organs and tissues (8).

Rheumatoid Arthritis (RA) is one of the most prevalent autoimmune diseases with unknown etiology. During the last decade many rheumatic syndromes have been related to bacterial, mycobacterial, viral, and fungal infections (9) and consequently, the importance of parasitic infections as underlying causes of rheumatic syndromes will likely grow as well. According to our data there is no research performed about the role of *Acanthamoeba* in producing RA or other autoimmune diseases in Iran.

This preliminary study was conducted to detect the prevalence of *Acanthamoeba* antibodies in RA patients and healthy controls to analyze the correlation between these two groups.

## Materials and Methods

### Sample sites and culture of Acanthamoeba

*Acanthamoeba* isolates were obtained from the various stagnant water resources in Tehran, Iran. From each sample 100–500 ml were vacuum-filtered through a 0.45 µm pore size. The filters were cultivated monoxenically in non-nutrient agar seeded with *Echerichia coli*. These plates were incubated at 37°C for 72 hours and monitored for growing of *Acanthamoeba* microscopically, then if necessary were kept for 2 weeks. *Acanthamoeba* trophozoites were identified by the presence of contractile vacuoles in cytoplasm and spiny surface projections called acanthopodia. *Acanthomoeba* cysts were identified with having a double-walled (ectocyst and endocyst) wrinkled approximately ranges in size from 13 to 20 µm. The samples containing *Acanthamoeba* were then transferred into axenic cultures by placing the amoebae into PYG medium (0.75% proteose peptone (wt/vol), 0.75% yeast extract (wt/vol), and 1.5% glucose (wt/vol) (6).

### Serum samples

During 10 months from October 2006 to August 2007 a total of 121 serum samples from RA patients attending the Rheumatolgy Department at Shariati Hospital in Tehran were obtained and stored at -20°C until using indirect fluorescent-antibody test (IFAT). RA was diagnosed according to the American Collage of Rheumatology classification criteria (10). Meanwhile, 126 healthy controls with no previous history of RA and other autoimmune diseases and without using of cytotoxic drugs matched with respect to gender and age. Study patients had a median age of 46.9±12.5 yr and healthy cases had 46.8 ±12.2 yr. Females made up 81% in patients group and 73% of healthy, respectively.

All serum samples were examined by indirect fluorescent-antibody tests (4) using a Nikon 80i microscope. Sample collection for this study was approved by the Ethic Committee of Research Division of Shahid Beheshti University (M.C). An informed consent was taken from all enrolled subjects.

### DNA extraction and PCR

To confirm the identity of *Acanthamoeba*, Polymerase Chain Reaction was performed using genus-specific primers as previously described (11). Approximately 10^3^ trophozoites (counted using a haemocytometer) from axenic culture were washed three times in phosphate – buffered salin (pH 7.4) and total DNA was extracted using a DNG™- Plus kit according to the manufacturer's instructions (Cinnagen, Tehran, Iran). Extracted DNA was stored at −20°C until use. To amplify of 18S rRNA gene a PCR protocol was used to amplify a fragment of 904 bp by using one set of oligonucleotide primers: 5'-GCTCCAATAGCGTATATTAA-3' and 5'-AGAAAGAGCTATCAATCTGT-3'.

The PCR mixture contained 1X PCR buffer, 4 mM MgCl_2_, 0.2 mM dNTP, 1.25 U *Taq* polymerase (Cinnagen, Iran), and 1 µM of each forward and reverse primers in a 25 µl reaction volume. Each of the 30 cycles consisted of 94°C for 30 s, 48°C for 30 s, and 72°C for 1 min after an initial denaturing at 94°C for 5 min and an extension at 72°C for 5 min. The PCR products were visualized by gel electrophoresis using 1.5% agarose, and the results were recorded on a UV gel documentation system (Cinnagen Ltd, Tehran, Iran).

## Results

### Culture

Out of 22 samples obtained from various sites in Tehran, 13 (59%) isolates readily adapted to growth in monoxenic medium and identified as *Acanthomoeba* spp. microscopically. Only two isolate were successfully cultured in an axenic (bacteria-free) medium. The antigen needed for IFAT obtained from these last isolates.

### PCR

**Fig. 1:**
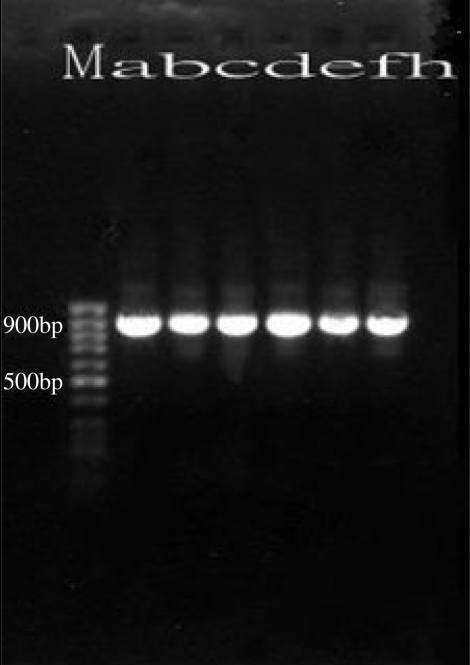
Gel electrophoresis of PCR products: M: 100 bp Marker;. a-f: *Acanthamoeba* sp; h: negative control

By using of 18S rRNA gene-based PCR assay, only six positive culture isolates were diagnosed as *Acanthamoeba* spp. The primers produced a fragment of 904bp, specific for identification of this genus (Fig. 1).

### Patients

**Fig. 2:**
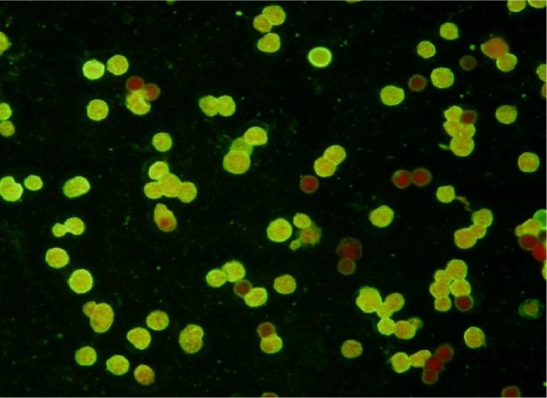
Indirect immunofluorescence for detection of *Acanthamoeba* specific antibodies (×200)

By IFA test (Fig. 2), *Acanthamoeba* antibodies were found in 127 of all 247 serum samples tested, with titers ranging from 1:10 to 1:320 (Table 1). The specific antibodies were detected in 70(57.8%) and 52(41.2%) of RA patients and controls, respectively. This difference was statistically significant (*P*<0.005). We detected no differences between gender and the presence of *Acanthamoeba* antibodies. By using class-specific labeled anti-immunoglobulins, it was shown that the antibodies belonged to IgG and IgM classes are more prevalent in patients than controls (respectively P<0.019 & P<0.003) (Table 2). The highest titer of total antibodies (1:320) was detected in one patient with RA.

**Table 1:** Seroprevalence of *Acanthamoeba* antibody titers in 121 RA patients and 126 controls

	<1/10	1/10	1/20	1/40	1/80	1/160	1/320	Total
RA patients n (%)	5 (4.1)	20 (16.5)	23 (19)	10 (8.3)	7 (5.8)	4 (3.3)	1 (0.8)	70 (57.8)
Controls n (%)	19 (15)	9 (7.1)	10 (7.9)	3 (2.4)	8 (6.3)	3 (2.4)	–	52 (41.3)
Total n (%)	24(9.7)	29 (11.7)	33 (13.4)	13 (5.3)	15 (6.1)	7 (2.8)	1 (0.4)	122 (49.4)

**Table 2:** Seroprevalence of IgG & IgM anti-*Acanthamoeba* in RA patients and controls

	IgG	IgM
RA patients; n (%)	33 (27.3)	30 (24.8)
Controls; n (%)	19 (15.1)	13 (10.3)

## Discussion

Our study showed that specific antibodies to *Acanthomoeaba* are common in both RA patients and healthy controls but we found that these antibodies were significantly more prevalent in RA patients (57.8%) in comparison with controls (41.2%) (P<0.005). This finding is on the contrary to another study in Norway, which found no significant difference of specific *Acanthomoeba polyphaga* antibodies between RA patients (86%) and controls (83%) (12). In another similar investigation in New Zealand, Cursons et al. detected the specific antibodies to *Acanthomoea* spp. in all 93 serum samples that were tested by IFAT^13^. In Czechoslovakia, where the sera of asymptomatic human were examined for *Acanthomoea* antibodies, the percentage of seropositivity varied from 3% in soldiers and psychiatric patients to 52% in hepatitis patients (14). Although our finding showed that the presence of both IgM and IgG antibodies were significantly higher in RA patients compared with controls, in some articles a lower frequency of IgG antibodies have been reported in RA patients compared with controls, this might be due to persistent or repeated antigenic stimulation induced by cysts, because under some conditions such as starvation, desiccation or extreme temperature, the trophozoite become encysted in a cellulose wall and survive for months or even as long as 24 years (15), a phenomenon such as toxoplasmosis (16, 17). In other hand, the widespread distribution of *Acanthamoeba* spp. in nature can be led to frequent exposure to this organism (18), but surprisingly most authors agree with this fact that despite high prevalence of specific antibodies in general population, the clinical cases have been reported rarely (19). Although some authors have recommended IFAT as a sensitive test for detection of specific antibodies of *Acanthomoeba* (20) it seems that, some limitation in titer reading may be responsible for the prevalence differences. Therefore, we are intending to assess our results by using a quantitative serological test such as ELISA.

With regard to our study, it seems that among parasite –related rheumatic syndromes that are largely unmentioned in medical textbook (21, 22) RA is one of the important syndromes that should be studied concisely. After reviewing of parasitic-related rheumatic diseases concluded that, often some sporadic cases had been reported and parasite was not mentioned in differential diagnosis of rheumatic syndrome.

In conclusion, because parasitic diseases are prevalent in most parts of Iran and increased cases of opportunistic infections in immunocompromised patients, *Acanthamoeba* must be considered as a pathogenic organism in this group.
